# Activation of Glycolysis by MCM10 Increases Stemness and Paclitaxel Resistance in Gastric Cancer Cells

**DOI:** 10.5152/tjg.2023.23169

**Published:** 2023-11-01

**Authors:** Zhangqiang Wu, Yuejun Fang, Jun Wu, Jianjun Wang, Yingjie Ling, Tao Liu, Qin Tong, Yefeng Yao*

**Affiliations:** Department of Surgical Oncology, Guang Fu Oncology Hospital, Jinhua, Zhejiang Province, China

**Keywords:** MCM10, glycolysis, cell stemness, gastric cancer, paclitaxel resistance

## Abstract

**Background/Aims:**

Chemotherapy is an essential avenue for curing malignancies; however, tumor cells acquire resistance to chemotherapeutic agents, eventually leading to chemotherapy failure. At present, paclitaxel (PTX) resistance seriously hinders the therapeutic efficacy of gastric cancer (GC). Investigating the molecular mechanism of PTX resistance in GC is critical. This study attempted to delineate the impact of MCM10 on GC resistance to PTX and its mechanism in GC.

**Materials and Methods:**

The expression of minichromosome maintenance complex component 10 (MCM10) in GC tissues, its enrichment pathways, and its correlation with glycolysis marker genes and stemness index (mRNAsi) were analyzed in a bioinformatics effort. Real-time quantitative polymerase chain reaction was used to assay the expression of MCM10 in cells. Cell counting kit-8 (CCK-8) was used to analyze cell viability and calculate the 50% inhibitor concentration (IC_50)_ value. Western blot was used to measure the expression of MCM10, Hexokinase 2 (HK2) and stemness-related factors in cells. Sphere-forming assay was performed to study cell sphere-forming ability. Seahorse XF 96 was utilized to measure cell extracellular acidification and oxygen consumption rates. The content of glycolysis-related products was tested with corresponding kits.

**Results:**

MCM10 was significantly upregulated in GC and enriched in the glycolysis pathway, and it was positively correlated with both glycolysis-related genes and stemness index. High expression of MCM10 increased sphere-forming ability of drug-resistant cells and GC resistance to PTX. The stimulation of PTX resistance and drug-resistant cell stemness in GC by high MCM10 expression was mediated by the glycolysis pathway.

**Conclusion:**

MCM10 was upregulated in GC and drove stemness and PTX resistance in GC cells by activating glycolysis. These findings generated new insights into the development of PTX resistance in GC, implicating that targeting MCM10 may be a novel approach to improve GC sensitivity to PTX chemotherapy.

**Main Points**

- High expression of MCM10 hindered gastric cancer (GC) cell sensitivity to paclitaxel (PTX).
- MCM10 facilitated GC resistance to PTX through the glycolysis pathway.
- MCM10 increased stemness and PTX resistance in GC cells via the glycolysis pathway.

## Introduction

Gastric cancer (GC) stands in the fifth place concerning the prevalence of malignancies and is the third main source of cancer-associated death worldwide.^1^ Gastric cancer can be attributed to *Helicobacter pylori *infection, age, a diet high in salt, and an inadequate intake of fruits and vegetables.^2^ Currently, surgery, chemotherapy, radiotherapy, targeted therapy, and immunotherapy are predominant options for GC patients. Among them, chemotherapy remains an obligatory clinical treatment for advanced GC. Paclitaxel (PTX) is a widely used, first-line chemotherapeutic agent for varied cancers, including GC, on account of its favorable therapeutic effect.^3,4^ The anticancer mechanism of PTX differs from other anticancer drugs that bind microtubulin in that PTX drives microtubulin polymerization, blocks cell cycle, and prevents mitosis, thereby hindering cancer cell growth.^5^ But PTX resistance has largely limited the efficacy of chemotherapy and its clinical application. Therefore, enhancing our knowledge of mechanisms of chemotherapy resistance in GC cells helps develop new approaches to improve treatment outcomes of GC patients.

MCM10 is an activator of the origin of DNA replication and, together with proliferation markers, initiates DNA replication at the late G1 or early S phase, thus driving cell proliferation.^6,7^ MCM10 expression is markedly upregulated in cancer cells and facilitates tumor progression. Yang and Wang^8^ denoted that MCM10 drives breast cancer cell malignant behaviors via Wnt/β-catenin signaling. Cui et al^9^ presented that knockdown of MCM10 suppresses prostate cancer cell malignant phenotype. In addition, aberrant expression of MCM10 affects chemotherapy resistance of tumors and cell stemness. Sphere-forming capacity of PTX-resistant breast cancer stem-like cells is dramatically reduced with MCM10 knockdown.^10^ Bao et al^11^ reported that in patients who acquired lapatinib resistance, increased MCM10 is a poor prognostic factor. Therefore, we speculated that MCM10 may be associated with PTX resistance in GC and investigated the mechanism.

This study not only enriched the knowledge of PTX resistance mechanism of GC but also provided new ideas for finding avenues to treat GC patients.

## Materials and Methods

### Bioinformatics Analysis

We utilized data from The Cancer Genome Atlas, which provided information on GC mRNA expression data (normal: 32, tumor: 375). The differential mRNAs were obtained by differential analysis using edxgeR (|logFC| > 2, *P*adj < .05), and the target mRNA was identified by referring to the literature. Single gene set enrichment analysis (GSEA) was done on target gene. Analysis of the correlation between target gene and stemness index (mRNAsi) was completed.

### Cell Culture and Establishment of Paclitaxel-Resistant Cell Line

Gastric mucosal epithelial (GES-1) and GC cells (MGC803, AGS, and MKN-45) were accessed from BeNa Culture Collection (BNCC, Xinyang, China). MGC803 cells and MKN-45 cells were maintained in Roswell Park Memorial Institute (RPMI) 1640, AGS cells in F12K, and GES-1 cells in dulbecco's modified eagle medium-high (DMEM-H). Media were treated with 10% fetal bovine serum, 100 U/mL streptomycin, and 100 U/mL penicillin. Incubation conditions were 37℃ and 5% CO_2_.^12^

Paclitaxel-resistant cells were established by sequentially increasing PTX concentrations (Selleckchem, Houston, Texas, USA) (0, 0.5, 1.0, 1.5, 2.0, and 2.5 μg/mL). MKN-45 and AGS cells were maintained in PTX (1µg/L)-contained media, respectively.^12^ Cells were successively cultivated at increasing concentrations of 25% PTX every 2 weeks. Paclitaxel-resistant cells (MKN-45/PTX and AGS/PTX) were established at a PTX concentration of 100 µg/L under the same culture conditions as the parental cells.^12^

The glycolysis inhibitor 2-DG was purchased from Selleck Corporation (Houston, Texas, USA). The control group was treated with equal amounts of phosphate buffered saline (PBS).

### Cell Transfection

Lipofectamine 2000 (Thermo Fisher Scientific, Stoney Creek, CA, USA) was utilized to transfect sh-MCM10, pcDNA3.1-constructed oe-MCM10, and corresponding negative controls sh-NC and oe-NC (Ribobio, Guangzhou, China) into GC cells. After incubating the cells for 24 hours, subsequent experimental measurements were conducted. Real-time quantitative polymerase chain reaction (qRT-PCR) and western blot were used to detect the efficiency of transfection.

### Real-Time Quantitative Polymerase Chain Reaction

Total RNA extraction from GC cells employed TRIzol reagent (Invitrogen, Carlsbad, CA, USA). A micro spectrophotometer was used to test the concentration of RNA. RevertAid First Strand cDNA Synthesis Kit (Thermo Fisher Scientific) was utilized to synthesize cDNA with RNA as the template. SYBR Premix Ex TaqII (Maokang Biotech, Beijing, China) was utilized to carry out qRT-PCR. β-actin was internal reference. Gene expression was calculated by 2^–ΔΔCt^. Primer information is mentioned in Table 1.

### CCK-8

CCK-8 kit (Biosharp, Hefei, China) was utilized to assess GC cell viability. After 0, 24, 48, and 72 hours of cell culture, 10 µL CCK-8 solution was added, respectively. Cells were subject to 2-hour-incubation at 37℃. The optical density (OD) value was assayed at 450 nm to reflect cell viability.

IC_50_ values of GC cells to PTX were assayed with CCK-8 kit (Biosharp). After incubating the transfected cells with different concentrations (0, 0.5, 1, 1.5, 2.0, and 2.5 µg/mL) of PTX for 24 hours, 10 µL of CCK-8 solution was added. The cell OD value was assayed at 450 nm to reflect cell viability, and the IC_50_ value was calculated.^12^

### Western Blot

Cultured cells were treated by PBS rinses, and total proteins were isolated with RIPA lysis buffer (Millipore, Boston, Massachusetts, USA) plus the protease inhibitor Cocktail (Roche, Basel, Switzerland) and the phosphatase inhibitor Cocktail 1/2 (Sigma-Aldrich, Saint Louis, MO, USA). Bio-Rad DC Protein Assay Kit (Bio-Rad, Hercules, CA, USA) was utilized for concentration quantification. Samples of 10-15 μg were separated by 10% Sodium Dodecyl Sulfate PolyAcrylamide Gel Electrophoresis (SDS-PAGE) and then transferred to polyvinylidene fluoride (PVDF) membranes, which were sealed with 5% skim milk in a buffer containing 1% Tween for 1 hour. Cell membranes were rinsed and incubated with primary antibodies specific for selected proteins [rabbit anti-human MCM10 (ab3733, 1: 1000), OCT4 (ab200834, 1:10 000), NANOG (ab109250, 1:1000), SOX2 (ab92494, 1:1000), HK2 (ab209847, 1:1000), and β-actin (ab8226, 1:1000)] overnight at 4°C, followed by peroxidase-coupled secondary antibody (goat anti-rabbit IgG, ab6721, 1:2000). Specific reactive proteins were tested with enhanced chemiluminescence (Pierce, Pierce, Missouri, USA). Antibodies were accessed from Abcam (Cambridge, UK).^13^

### Tumor Sphere-Formation Assay

Cells were trypsinized and seeded (500 cells/well) on the ultra-low attachment 6-well plates. RPMI 1640/F12K serum-free medium (Invitrogen) was added with 2% B-27 (Invitrogen), 0.4% BSA (Sigma-Aldrich), fibroblast growth factor (20 ng/mL; Sigma-Aldrich), epidermal growth factor (20 ng/mL; Sigma-Aldrich), and insulin (5 μg/mL; Sigma-Aldrich). Cells were maintained at 37℃ and 5% CO_2_ for 11 days. Diameters of the spheroids were subsequently counted under the microscope.^14^

### Glucose Metabolism Assay

The impact of overexpression of MCM10 and 2-deoxy-d-Glucose (2-DG) inhibitor on cellular extracellular acidification rate (ECAR) and oxygen consumption rate (OCR) was examined by XF 96 extracellular flux analyzer (Agilent Technologies, Santa Clara, CA, USA). Briefly, 1 × 10^4^ cells were inoculated into 96-well Seahorse plates and maintained overnight at 37℃. About 2.5 μM oligomycin, 0.25 μM rotenone, and 2 μM FCCP were added for OCR (pmol /min) detection. Extracellular acidification rate (mpH/min) was measured after the addition of 10 mM glucose, 50 mM 2-DG, and 1 μM oligomycin. Oxygen consumption rate and ECAR curves were normalized according to protein concentration and plotted on Wave software (Agilent Technologies).^15^

### Determination of Content of Glycolysis Products

The contents of lactate, pyruvic acid, citrate, and malate were determined using the corresponding kits. The citrate assay kit was obtained from Solarbio (Beijing, China), and the rest of the assay kits were accessed from Biovision (Palo Alto, CA, USA).

### Statistical Analysis

GraphPad Prism 5 (GraphPad Software, San Diego, CA, USA) was applied for the analysis of data. Each experiment was repeated 3 times. The Mann–Whitney *U* test was utilized for 2-group comparisons because the sample size was small and the normal distribution assumption was invalid. The Kruskal–Wallis test was utilized to determine the statistical significance of multiple comparisons. *P* < .05 denoted a statistically significant difference.

## Results

## MCM10 is Upregulated in Gastric Cancer

**Figure 1.**
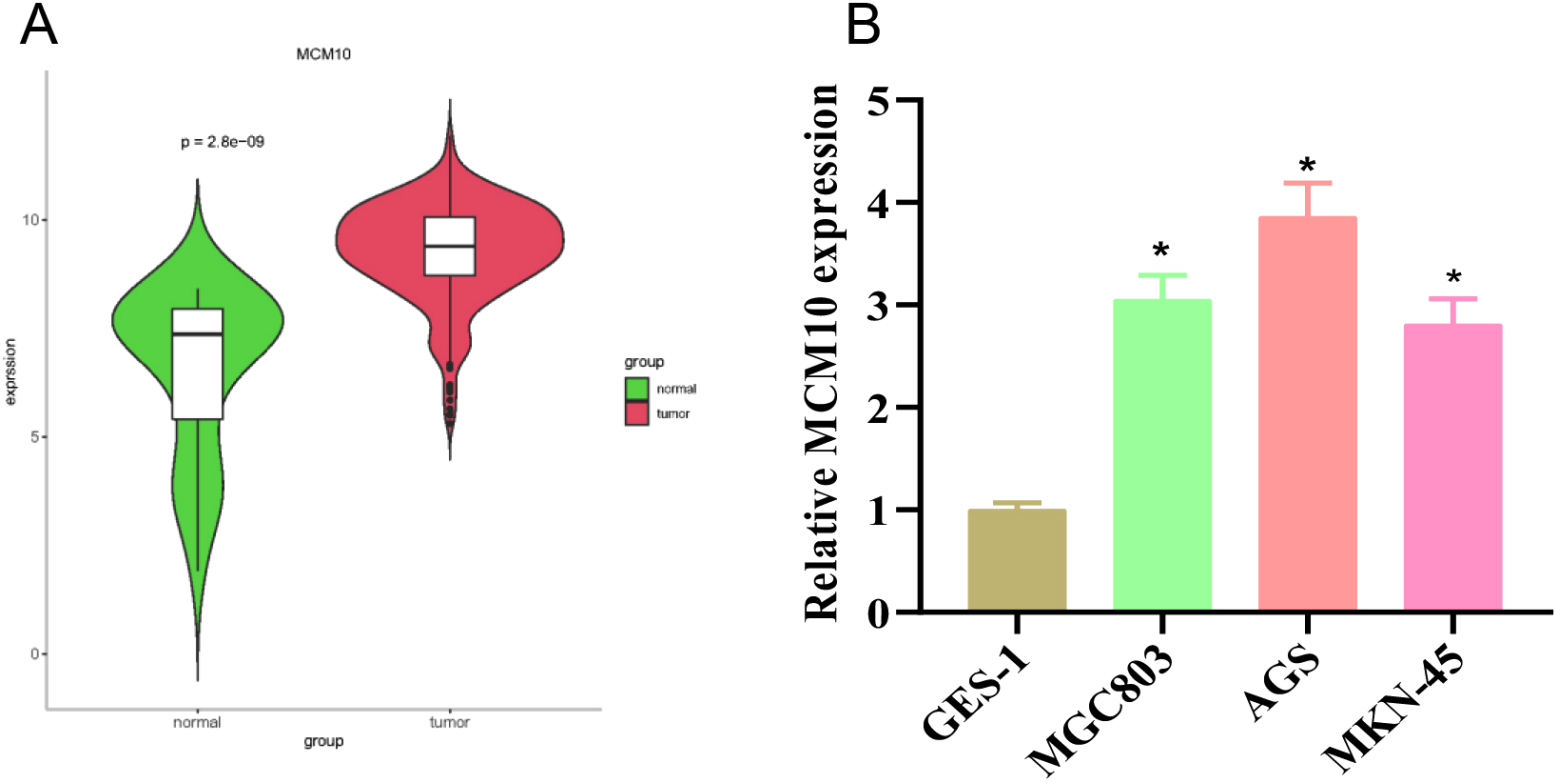
MCM10 is upregulated in gastric cancer (GC). (A) MCM10 expression in GC tissues and normal tissues as predicted and analyzed through The Cancer Genome Atlas. (B) MCM10 expression in GC cells and gastric mucosal epithelial cells as assayed by real-time quantitative polymerase chain reaction. GES-1, gastric mucosal epithelial. *P < .05.

MCM10 is highly expressed in ovarian cancer, prostate cancer, and breast cancers.^8,9,16^ MCM10 was significantly highly expressed in GC tissues as found by bioinformatics analysis (Figure 1A). MCM10 expression in human GES-1 cells and GC cells was assessed by qRT-PCR. Data suggested that compared with GES-1 cells, MCM10 expression was significantly upregulated in GC cells (Figure 1B). These findings revealed the significantly high expression of MCM10 in GC. The expression level of MCM10 was the highest in AGS cells, while the expression level of MCM10 was the lowest in MKN-45. Therefore, AGS cells were selected for knockdown treatment, while MKN-45 cells were overexpressed.

### Effect of Abnormal MCM10 Expression on Gastric Cancer Sensitivity to Paclitaxel

**Figure 2.**
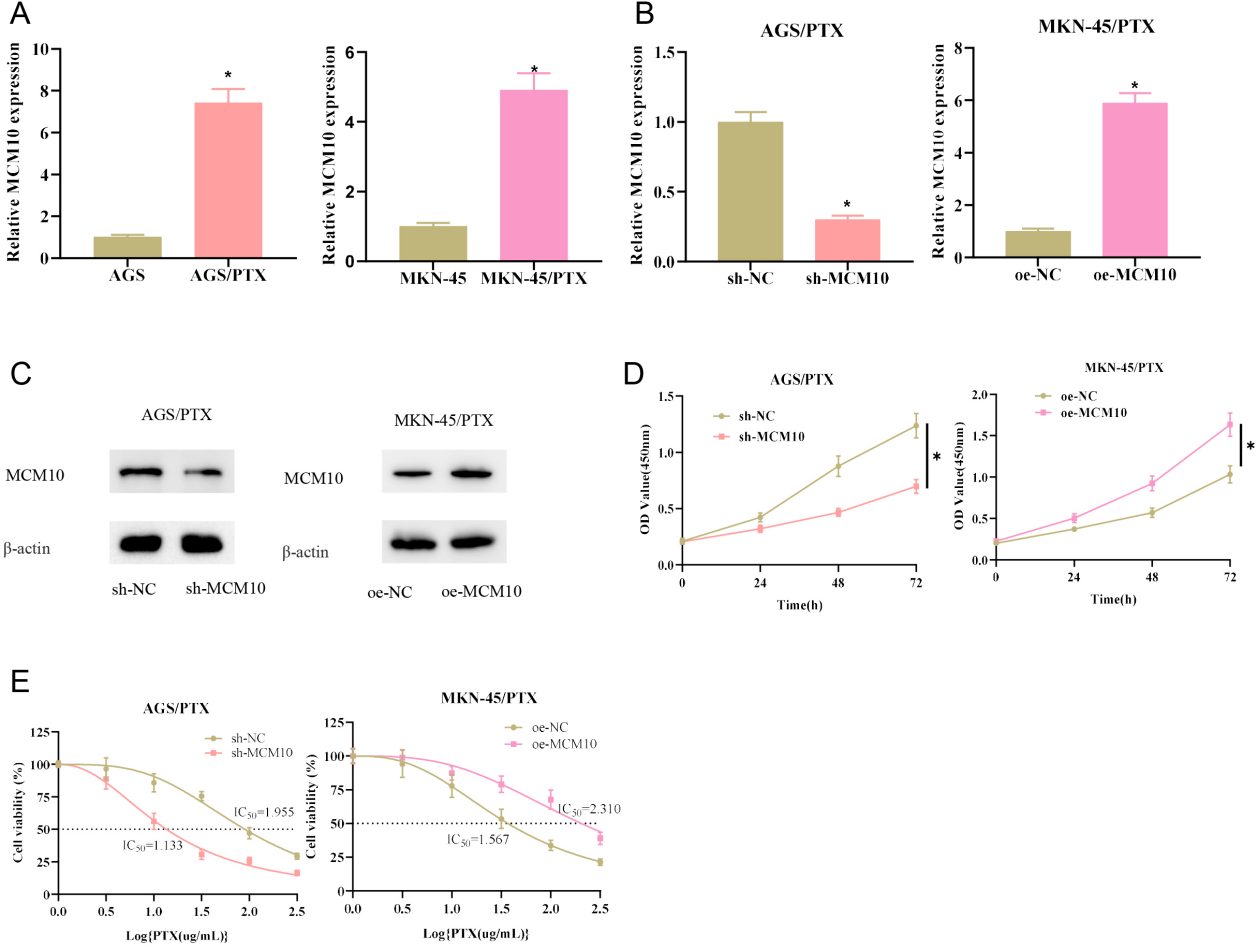
Effect of abnormal MCM10 expression on gastric cancer (GC) sensitivity to paclitaxel (PTX). (A) MCM10 expression in GC-resistant and non-resistant cells. (B-C): The effects of knockdown and overexpression of MCM10 on MCM10 expression in drug-resistant cells were tested by real-time quantitative polymerase chain reaction and western blot. (D) The effects of knockdown of MCM10 and overexpression of MCM10 on the viability of drug-resistant cells. (E) The effects of sensitivity of the drug-resistant cells to PTX in each treatment group. *P < .05.

MCM10 is increased in breast cancer and essential for cell proliferation and maintenance of PTX-resistant cancer stem-like cells.^10^ This finding led us to propose the hypothesis that high expression of MCM10 in GC affected GC cell resistance to PTX. To examine our hypothesis, we selected AGS cells with the highest level of MCM10 expression and MKN-45 cells with a relatively low expression to establish PTX-resistant cells. MCM10 expression was detected in GC-resistant and sensitive cells using qRT-PCR. The data presented that MCM10 was significantly highly expressed in AGS/PTX and MKN-45/PTX cells compared with sensitive cells (Figure 2A). To investigate the relationship of MCM10 with PTX resistance in GC, sh-MCM10/ sh-NC plasmids were transfected into AGS/PTX cells and oe-MCM10/ oe-NC plasmids into MKN-45/PTX cells. Transfection efficiency of each treatment group was examined. Treatment with sh-MCM10 significantly decreased MCM10 expression in AGS/PTX cells, and oe-MCM10 significantly increased MCM10 in MKN-45/PTX cells (Figure 2B and 2C). The results of CCK-8 as depicted in Figure 2D presented that sh-MCM10 substantially decreased the viability of AGS/PTX cells, but oe-MCM10 substantially increased the viability of MKN-45/PTX cells. The effect of aberrant MCM10 expression on PTX sensitivity in GC-resistant cells was then examined. The experimental data suggested that sh-MCM10 markedly reduced the IC_50_ value of AGS/PTX cells and oe-MCM10 markedly elevated the IC_50_ value of MKN-45/PTX cells (Figure 2E). Taken together, the findings demonstrated that MCM10 was increased in GC-resistant cells and increased GC cell resistance to PTX in vitro.

### MCM10 High Expression Increases Stemness in Paclitaxel-resistant Gastric Cancer Cells

**Figure 3.**
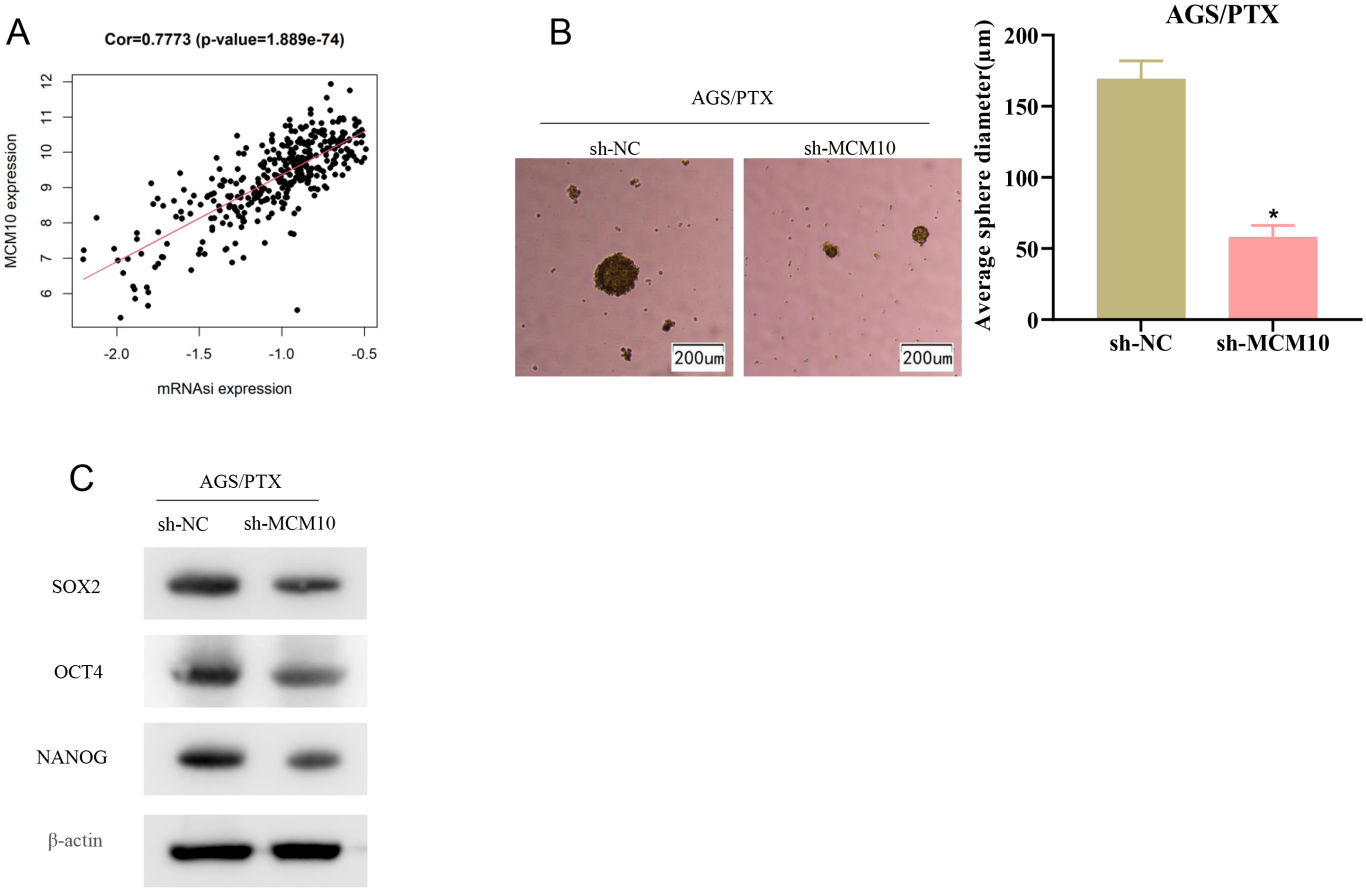
MCM10 high expression increases cell stemness in paclitaxel-resistant gastric cancer cells. (A) Correlation analysis of MCM10 with stemness index. (B) Sphere-forming ability of drug-resistant cells after transfection. (C) Protein expression of stem cell markers after transfection. Cor, correlation; mRNAsi, stemness index; PTX, paclitaxel. *P < .05.

Enhanced cell stemness drives chemotherapy resistance in tumor cells.^14^ Correlation analysis of MCM10 with mRNAsi unveiled a significant positive correlation (Figure 3A). sh-MCM10/sh-NC drug-resistant cell groupings were established based on AGS/PTX cells. Sphere-forming ability was examined by cell sphere-forming assay. Knockdown of MCM10 notably repressed the formation of AGS/PTX-based tumor spheroids (Figure 3B). Stem cell marker (OCT4, NANOG, and SOX2) expressions were detected at protein level. These protein expression levels were measurably significantly downregulated (Figure 3C). To sum up, MCM10 high expression enhanced stemness in PTX-resistant GC cells.

### MCM10 High Expression Enhances Gastric Cancer Cell Stemness and Paclitaxel Resistance Through the Glycolysis Pathway

**Figure 4.**
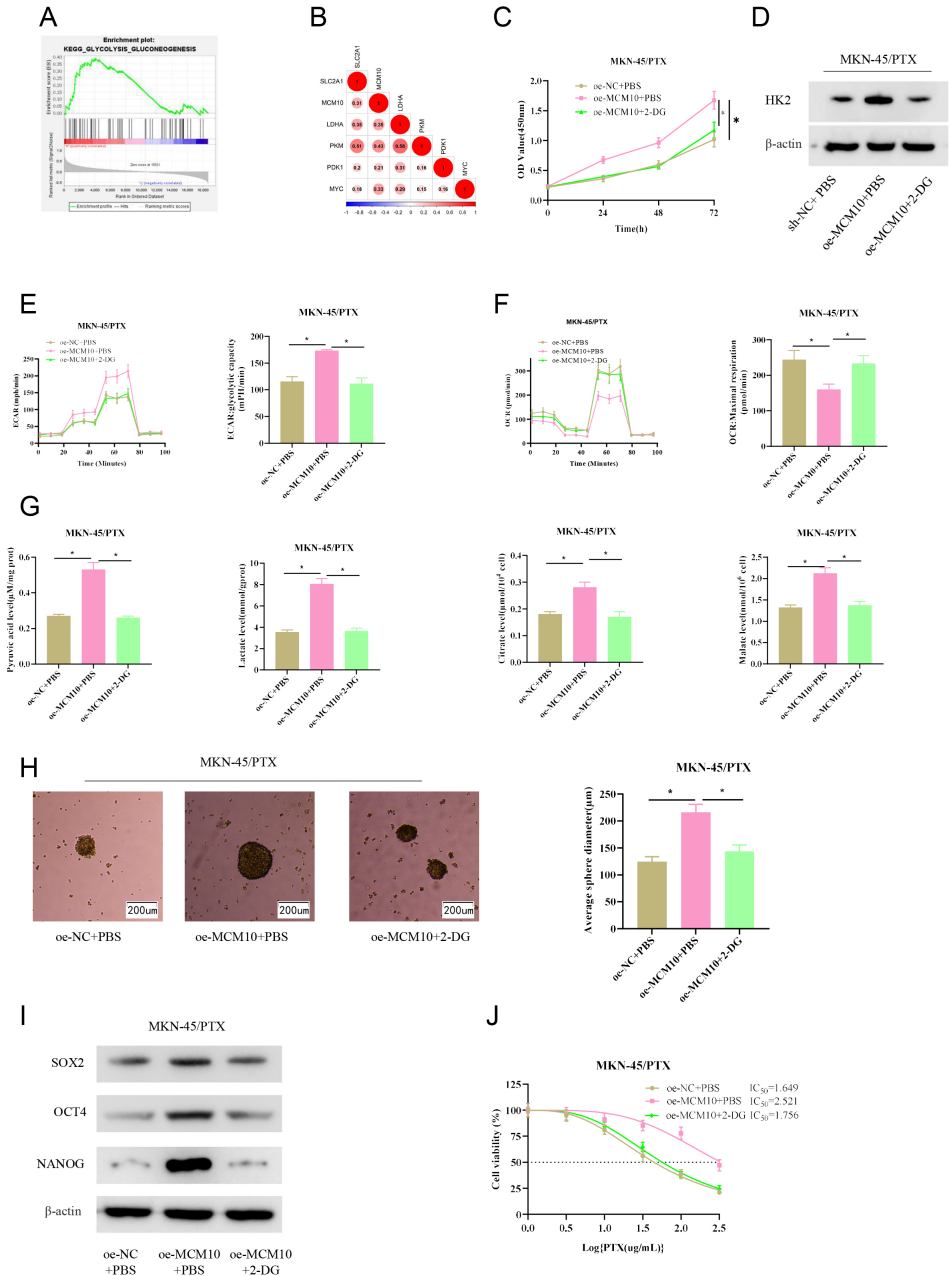
MCM10 high expression increases gastric cancer cell stemness and paclitaxel resistance through the glycolysis pathway. (A) Gene set enrichment analysis results of MCM10. (B) Correlation analysis of MCM10 with glycolysis-related genes. (C) Cell viability in each group was tested by the CCK-8 assay. (D) HK2 protein expression in cells in each group was tested by western blot assay. (E, F) Extracellular acidification rate and oxygen consumption rate in each group. (G) The contents of glycolysis products in each group were tested by ELISA kit. (H) Sphere-forming ability of cells in each group was tested by sphere-forming assay. (I) The expression of stem cell markers at the protein level was tested by western blot assay. (J) The IC_50_ value of cells in each group was tested by the CCK-8 assay. ECAR, extracellular acidification rate; GSEA, gene set enrichment analysis; GC, gastric cancer; OCR, oxygen consumption rate; PTX, paclitaxel. *P < .05.

To further dissect the mechanism by which MCM10 affected GC cell stemness and PTX resistance, single GSEA was conducted on MCM10, which was significantly enriched in the glycolysis pathway (Figure 4A). The correlations of MCM10 with glycolysis marker genes PDK1, LDHA, PKM, MYC, and SLC2A1 were analyzed, and significant positive correlations were revealed (Figure 4B). To elucidate the influence of high MCM10 expression on glycolysis, we transfected MKN-45/PTX cells using oe-MCM10 and 2-DG inhibitors and divided them into oe-NC+PBS control group, oe-MCM10+PBS group, and oe-MCM10+2-DG group. CCK-8 assay unraveled that oe-MCM10 noticeably enhanced the viability of MKN-45/PTX cells, and transfection of 2-DG inhibitor reversed this effect (Figure 4C). Western blot assayed the expression of glycolysis-related protein HK2, and the data suggested that overexpression of MCM10 upregulated the expression of HK2, which was reversed by treatment with 2-DG inhibitor (Figure 4D). Data from the Seahorse XF 96 instrument showed that overexpression of MCM10 substantially enhanced ECAR and reduced OCR, while concurrent treatment with 2-DG inhibitor reversed these effects (Figure 4E and 4F). Additionally, the overexpression of MCM10 also drove an increase in the content of glycolysis products, and concomitant treatment with 2-DG inhibitor reversed this result (Figure 4G). The view that aerobic glycolysis facilitates tumor cell stemness^17^ prompted us to propose the hypothesis that high expression of MCM10 in GC may increase cell stemness through glycolysis. Cell sphere-forming assay revealed that forced expression of MCM10 markedly enhanced sphere-forming capacity of MKN-45/PTX cells, but the addition of 2-DG inhibitor rescued sphere-forming ability to the control level (Figure 4H). Western blot detection of OCT4, NANOG, and SOX2 protein levels in cells in each group presented that the expression was dramatically upregulated, but the addition of 2-DG inhibitor reversed the effect (Figure 4I). Finally, the influence of glycolysis on GC cell resistance to PTX was examined. CCK-8 assay unveiled that oe-MCM10 elevated the IC_50_ value of MKN-45/PTX cells, and concurrent treatment with 2-DG inhibitor reversed this effect (Figure 4J). The above findings demonstrated that high MCM10 expression enhanced stemness and PTX resistance in GC cells by activating glycolysis.

## Discussion

Gastric cancer is a prevalent malignancy globally, and patients with advanced GC have a 5-year survival rate of around 20%.^18^ Chemotherapy is the mainstay of GC treatment, but drug resistance is the biggest deterrent to the effectiveness of chemotherapy.^19^ Paclitaxel, the main chemotherapeutic agent for GC, represses mitosis and induces apoptosis through microtubule stabilization,^20^ but the molecular mechanism by which resistance develops is still uncharacterized. Hence, the underlying mechanism of PTX resistance is in urgent need of elucidation, which is important for the development of therapeutic agents to reverse GC chemoresistance. We found that the expression level of MCM10 in PTX-resistant strains was significantly higher than that in non-resistant cell lines, indicating that PTX resistance may be related to the abnormal expression of MCM10. However, the mechanism by which MCM10 affects PTX resistance in GC cells still needs further research.

In contrast to the surrounding tissues, even with oxygen, malignant solid tumors need an abundance of glucose to generate lactate and ATP, a phenomenon known as aerobic glycolysis,^21^ which is a distinguishing sign of cancer. Increased glycolysis can provide energy for tumor cell growth and proliferation.^22^ Previous studies have confirmed that aerobic glycolysis affects tumor progression. Robinson et al^23^ demonstrated that NOX2 in acute myeloid leukemia (AML) cells can promote glycolysis by activating PFKFB3, thus driving the proliferation of AML cells. Li et al^24^ confirmed that 100A2 induces glycolysis reprogramming through the upregulation of glucose transporter-1 (GLUT1) expression via the phosphoinositide 3 kinase/Protein Kinase B (PI3K/AKT) pathway, thus driving cell proliferation and progression in colorectal cancer. Additionally, glycolysis influences cancer cell drug resistance, and drug-resistant cells are more glycolytic and produce more adenosine-triphosphate (ATP).^25^ Artesunate (ART) enhances adriamycin (ADR) toxicity by suppressing glycolysis, mdr1/abcg2 level in K562/ADR cells.^26^ Wang et al^25^ ascertained that exosomes originating from CRC cells resistant to oxaliplatin transfer ciRS-122 to sensitive cells and enhance glycolysis by increasing the PKM2 expression to enhance oxaliplatin resistance. Our data suggested that MCM10 was enriched in the glycolysis pathway and highly expressed in GC, and it facilitated glycolysis metabolism, thereby inducing PTX resistance in GC cells. Meanwhile, we found that low expression of MCM10 could repress the cell ability. It indicated that MCM10 acted as an oncogenic gene in GC cells. In the qRT-PCR assay, we found that the expression level in Figure 1B was inconsistent with that in Figure 2A; the reason may be that the qRT-PCR results were relatively quantitative. Our study revealed a novel mechanism of MCM10 in GC PTX resistance, offering a new perspective to improve the efficacy of GC chemotherapy.

Tumor cell metabolic reprogramming exerted an essential modulatory role in cancer stem cells. Labeled tumor-initiating cells, these cells are quiescent, multipotent, and self-renewing tumor cells.^27^ Besides, they are a fraction of tumor cells that can initiate tumorigenesis and cause tumor recurrence.^28^ Tumor cell aerobic glycolysis is closely linked to cancer stem cell properties. Mori et al^29^ revealed that acetaldehydedehydrogenase increases glycolysis through the upregulation of GLUT1 and enhances cell stemness in endometrial cancer. Yang et al^30^ unraveled that enolase 1 (ENO1) facilitates GC cell stemness by enhancing glycolytic capacity of cells. We demonstrated similar findings that MCM10 increased cell stemness in GC by activating the glycolysis pathway and that 2-DG inhibitor was able to reverse the stimulation of cell stemness by overexpression of MCM10. In summary, we elucidated the specific mechanism by which high expression of MCM10 affected GC cell stemness, suggesting that repression of glycolysis pathway may be a new approach to hamper GC cell stemness, and targeting MCM10 may be a promising target to mitigate GC cell stemness.

Taken together, our data confirmed a significantly high expression of MCM10 in GC. Mechanistically, our current findings presented that the overexpression of MCM10 enhanced cell stemness of PTX-resistant cells and GC resistance to PTX by a mechanism mediated through the glycolysis pathway. These results was investigated at the cellular levels, where lies the shortcoming of this study that they have not yet been validated by *in vivo* animal experiments. The ongoing subject is to dissect further the influence of differential expression of MCM10 on tumor growth and its resistance to PTX in mice by constructing a GC mouse xenograft model. Overall, MCM10 may be a biological target to mitigate GC chemoresistance and provide a new direction to reverse PTX resistance in GC therapy.

## Figures and Tables

**Table 1.** The Real-time Quantitative Polymerase Chain Reaction Primer Sequences

Gene	Forward Primer	Reverse Primer
MCM10	5’-GAAGAAGGTTACGCCACAGAG-3’	5’-TTTACAGGTTCCCAGGTCAAG-3’
β-actin	5’-AGAAGGCTGGGGCTCATTTG-3’	5’-AGGGGCCATCCACAGTCTTC-3’
